# Movement Interferes with Visuospatial Working Memory during the Encoding: An ERP Study

**DOI:** 10.3389/fpsyg.2017.00871

**Published:** 2017-05-29

**Authors:** Rumeysa Gunduz Can, Thomas Schack, Dirk Koester

**Affiliations:** ^1^Neurocognition and Action – Biomechanics Research Group, Faculty of Psychology and Sport Science, Bielefeld UniversityBielefeld, Germany; ^2^Cognitive Interaction Technology – Center of Excellence, Bielefeld UniversityBielefeld, Germany; ^3^Research Institute for Cognition and Robotics, Bielefeld UniversityBielefeld, Germany

**Keywords:** ERPs, dual-task interference, manual actions, verbal working memory, visuospatial working memory, encoding processes, retrieval processes

## Abstract

The present study focuses on the functional interactions of cognition and manual action control. Particularly, we investigated the neurophysiological correlates of the dual-task costs of a manual-motor task (requiring grasping an object, holding it, and subsequently placing it on a target) for working memory (WM) domains (verbal and visuospatial) and processes (encoding and retrieval). Thirty participants were tested in a cognitive-motor dual-task paradigm, in which a single block (a verbal or visuospatial WM task) was compared with a dual block (concurrent performance of a WM task and a motor task). Event-related potentials (ERPs) were analyzed separately for the encoding and retrieval processes of verbal and visuospatial WM domains both in single and dual blocks. The behavioral analyses show that the motor task interfered with WM and decreased the memory performance. The performance decrease was larger for the visuospatial task compared with the verbal task, i.e., domain-specific memory costs were obtained. The ERP analyses show the domain-specific interference also at the neurophysiological level, which is further process-specific to encoding. That is, comparing the patterns of WM-related ERPs in the single block and dual block, we showed that visuospatial ERPs changed only for the encoding process when a motor task was performed at the same time. Generally, the present study provides evidence for domain- and process-specific interactions of a prepared manual-motor movement with WM (visuospatial domain during the encoding process). This study, therefore, provides an initial neurophysiological characterization of functional interactions of WM and manual actions in a cognitive-motor dual-task setting, and contributes to a better understanding of the neuro-cognitive mechanisms of motor action control.

## Introduction

The vast majority of interaction with the physical world is accomplished through manual actions. For example, we reach for objects at different distances, we grasp and lift objects with different shapes, weight and texture, and we manipulate objects depending on our goals while keeping our current tasks available [in working memory (WM)]. Planning and executing skilled manual actions require precise motor control which is provided by the integration of not only the sensory and motor systems, but also of the cognitive systems (Castiello, 2005; Olivier et al., 2007; Grafton, 2010). The present study focuses on the functional interactions of cognition and manual actions. Particularly, in a dual-task setting, we investigate the neurophysiological correlates of the functional interactions of WM and a manual-motor movement which includes grasping an object and placing it on a target.

The way how we interact with and manipulate an object is shaped by both the external and internal factors. External factors refer to both an object’s intrinsic physical properties such as size, shape and texture, and extrinsic physical properties such as location and orientation (Galletti et al., 2003; Castiello and Begliomini, 2008). In natural environments, manual actions are not performed solely based on the external factors. Indeed, they are performed based also on the internal factors which are mainly cognitive variables (for reviews, see Glover, 2004; Rosenbaum et al., 2012, 2014). For example, the goals of the agents, the intentions about the object use, familiarity with the object, and the affordances the object provides also shape the way how we interact with and manipulate objects (e.g., Creem and Proffitt, 2001; Tucker and Ellis, 2001, 2004; Grezes et al., 2003; Ansuini et al., 2008; Herbort and Butz, 2010, 2011). Think about a hand movement toward a fork. We would reach for and grasp the fork differently depending on whether we place it in a cupboard or use it for eating. Moreover, in the natural environments manual actions are often performed concurrently with other cognitive tasks. For example, we can engage in a conversation with a friend while interacting with the fork in that particular context. Therefore, considering the required sensory, motor and cognitive systems, it can be suggested that cognition and manual actions are not functionally independent from each other; rather there may be a cross-talk between them.

Working memory is one of the cognitive domains, besides, for example, language (e.g., Glover et al., 2004; Lindemann et al., 2006) or perception (e.g., Deubel et al., 1998), being shown to interact with manual actions. WM is the cognitive process which temporarily stores and manipulates information for coordinating various activities such as maintaining an action goal (grasping the fork to place it in the cupboard) and holding a conversation (Baddeley, 2003). WM and motor actions have a close functional interaction, which has been shown for different actions such as finger tapping (e.g., Smyth et al., 1988; Smyth and Pendleton, 1989), pointing (e.g., Hale et al., 1996), arm movements (e.g., Quinn and Ralston, 1986; Lawrence et al., 2001) as well as eye movements (e.g., Lawrence et al., 2001; Postle et al., 2006). WM for objects with affordances has been shown to depend on the activation of motor programs associated with object use, which in turn, reflects the involvement of motor processes in WM (e.g., Mecklinger et al., 2002, 2004). Moreover, WM processes have been shown to be involved in complex motor actions including sequences such as dance movements (e.g., Cortese and Rossi-Arnaud, 2010). WM processes are also employed during grasping movements, for example, to keep the goal relevant information for a subsequent execution of the grasp (e.g., Fiehler et al., 2011; Fournier et al., 2014). Accordingly, the execution of the grasping movements after a delay has been shown to depend on WM (e.g., Kohler et al., 1989; Binsted et al., 2006; Singhal et al., 2007; Hesse and Franz, 2010).

Cognitive-motor dual-task paradigms have been used to investigate the functional interactions of WM and motor actions (e.g., Voelcker-Rehage and Alberts, 2007; Weigelt et al., 2009; Logan and Fischman, 2011; Spiegel et al., 2012, 2013, 2014; Guillery et al., 2013). The logic of dual-task paradigms is that simultaneous performance of two tasks would result in interference between the tasks if both tasks compete for the same capacity-limited cognitive resources. This interference would be demonstrated by means of decreases in performance of either of the tasks or both (Pashler, 1994; Saling and Phillips, 2007; Wickens, 2008). Studies employing dual-task paradigms have shown that motor tasks requiring manual actions recruit the capacity-limited cognitive resources which are also required for performing a memory task. That is, concurrent motor tasks interfere with WM and result in memory performance decrease (e.g., Weigelt et al., 2009; Logan and Fischman, 2011; Spiegel et al., 2012, 2013, 2014). For example, Weigelt et al. (2009) combined a perceptual-motor task (opening a sequence of drawers to grasp cups in the drawers) with a verbal short-term memory task (recalling a sequence of letters positioned in the cups). By doing so, they investigated whether and how the motor planning and verbal short-term memory would interfere with each other. Weigelt et al. (2009) showed that the motor planning eliminated the recency effect, i.e., the tendency of recent items to be recalled better than earlier items in a list, which otherwise is a robust memory effect. The researchers interpreted this finding as suggesting that the motor planning recruits the capacity-limited cognitive resources which are also required for performing the (working) memory task. Logan and Fischman (2011), in a complex everyday task setup, showed that not only the motor activity requiring complex planning, but also the motor activity requiring no or limited planning could eliminate the recency effect.

Recently, Spiegel et al. (2013) investigated the functional interactions of WM and manual actions in a cognitive-motor dual-task paradigm taking two points into consideration. First, goal-directed actions have been suggested to consist of two functionally distinct components, i.e., planning and execution (online control), which rely on different perceptual and cognitive representations (Elliott et al., 2001; Glover, 2004). Second, WM has been suggested to have functionally distinct verbal and visuospatial domains, which are selectively interfered by motor actions (Baddeley and Hitch, 1974; Baddeley, 2003; Logie, 2011). Spiegel et al. (2013) first investigated the dual-task costs of performing a prepared movement for WM by combining verbal and visuospatial versions of a WM task with a manual-motor task which included the condition of grasping an object and placing it on a target position. They showed that performing a prepared movement (execution) interfered more with memorizing the visuospatial material compared with the verbal material and decreased the memory performance for the visuospatial WM task, i.e., domain-specific memory costs. Second, they investigated the dual-task costs of changing the plan of an ongoing movement (movement re-planning). Unlike the execution, (re-)planning of the movement interfered with memorizing both the verbal and visuospatial material to a similar degree and decreased the memory performance for both WM tasks, i.e., domain-general memory costs. These findings suggest that both movement components recruit distinct WM resources and therefore lead to unique interference with WM domains. While, the movement execution shares the capacity-limited cognitive resources mainly with the visuospatial domain, movement planning shares the cognitive resources with both domains.

As aforementioned, it has been shown that manual actions have a close functional interaction with WM, which is complex and dependent of variety of factors (e.g., Spiegel et al., 2013). However, there is still lack of research investigating the underlying cortical activity. Therefore, the goal of the present study is to extend the knowledge for WM and manual action interactions to the neurophysiological level. Specifically, the present study investigated the neurophysiological correlates of dual-task costs of a manual-motor task for WM. This motor task (requiring limited motor planning) included grasping an object, holding it, and then subsequently placing it on a target. Therefore, in the rest of the paper, we will use the term ‘grasping-and-placing movement’ descriptively to refer to the manual action required in this study.^1^

We adapted the cognitive-motor dual-task paradigm from the behavioral work by Spiegel et al. (2012, 2013, 2014) to the electroencephalogram (EEG) setting. This way, we aimed to replicate their findings and could use an established experimental paradigm for the present exploration of the underlying neurophysiological activity (EEG). In the baseline single-task condition, participants performed the verbal (recall of letters) or visuospatial (recall of symbols positioned in a 4 × 4 matrix) version of a WM task. In the dual-task condition, the WM task was embedded in a manual-motor task which included grasping-and-placing movement. To investigate the neurophysiological correlates of dual-task costs of the motor task, EEG data was recorded during the WM tasks both in single and dual-task conditions.

The present study aims to determine the source of dual-task costs of the motor task for WM domains. Depending on the function, WM has been suggested to have three cognitive processes (i.e., encoding, maintenance, and retrieval) which represent distinct cognitive operations of information in WM and arise from separate neural sources (e.g., Hale et al., 1996; Geffen et al., 1997; Manoach et al., 2003; Bledowski et al., 2006; Studer et al., 2010; Pinal et al., 2014). Encoding is the process during which a stimulus is perceived and a representation of it is generated. Maintenance is the process during which the stimulus must be retained active in memory when the perceptual input of the stimulus is not available. Retrieval is the process during which the stored information is accessed for performing the task at hand (Jonides et al., 2008). Given the distinct cognitive operations in encoding, maintenance and retrieval processes, the motor task could interfere with WM domains uniquely in each process.

With the aim of determining the source of the interference with WM domains, the present study made use of the event-related potentials (ERPs). ERPs are voltage fluctuations being extracted from EEG recordings in response to a cognitive or motor process (Hillyard and Kutas, 1983; Friedman and Johnson, 2000). ERPs have high temporal resolution in order of milliseconds, so they can be used to measure rapidly changing dynamics of any cognitive process (Pinal et al., 2014). ERPs also provide useful information about the scalp distribution of the neural activity related to different cognitive processes. That is, comparisons of the spatial distribution of ERPs elicited by different experimental conditions could indicate whether these conditions entail different patterns of cortical activity, thus, likely reflect different cognitive processes (Woodman, 2010). Therefore, ERPs are particularly suitable for studying spatio-temporal characteristics of transient operations independently in each WM domain and each WM process, i.e., encoding, maintenance and retrieval. This is feasible since each WM process shows stable time relationships to separately defined reference events, such as stimulus presentation (encoding process), offset of stimulus presentation (maintenance process) or recall (retrieval process) (Friedman and Johnson, 2000).

Consistently, EPRs also provide a reliable method for investigating the distinct motor task interference with each WM process, which would be very difficult to address only with behavioral data. The present study, therefore, investigated the WM processes during which the motor task interferes with WM, i.e., whether the interference arises during either of the encoding or retrieval of WM material or both (see Materials and Methods section for the reason we could not include maintenance process). For this aim, ERPs were analyzed separately for encoding and retrieval processes during verbal and visuospatial WM tasks both in single and dual-task conditions.

The present study has three objectives: First, we aim to replicate the findings of the behavioral study by Spiegel et al. (2013) in an EEG setting. Second, as our main objective, we aim to provide an initial neurophysiological characterization of the functional interactions of WM and manual actions by focusing on separate WM domains and processes. To the best of our knowledge, the present study is one of the few studies investigating the ERPs in an experimental setting requiring movement execution (e.g., van Schie and Bekkering, 2007; Westerholz et al., 2013, 2014) and the first study investigating the ERPs of the functional interactions of WM and manual actions in a dual-task setting. Therefore, in a more general level, the present study also aims to demonstrate whether reliable ERPs can be analyzed in complex experimental settings which involve movement execution such as grasping an object to place it on a target, and hand writing for reporting the WM items. This, in turn, should encourage future research options with more sensitive measures such as ERPs as opposed to the behavioral measures for the investigation of multiple cognitive processes in motor action control (e.g., Spiegel et al., 2012, 2013, 2014).

Regarding the behavioral analyses, we formulated our hypothesis based on the previous findings by Spiegel et al. (2013). We expected that concurrently performed prepared movement would interfere with WM and decrease the memory performance, which would be larger for the visuospatial task compared with the verbal task. That is, the prepared manual-motor movements are expected to entail domain-specific memory costs.

Regarding the ERP analyses, given a lack of comparable ERP studies investigating the WM-manual action interactions, we formulated our hypotheses based on the limited, available ERP findings on either WM or manual actions. To the best of our knowledge, most ERP studies have focused mainly on the maintenance process of WM, although the gaining a complete understanding of the cortical activity involved in WM requires dissociating cognitive processes of encoding, maintenance, and retrieval (Manoach et al., 2003; Jonides et al., 2008). Importantly, the present study required a joint processing of each WM process and grasping-and-placing movement. Therefore, we chose a starting point which could further provide hypotheses regarding the ERPs of WM-manual action interactions. We formulated our hypotheses regarding the WM-related ERPs based on widely reported slow waves for each WM domain: The (left) anterior negative slow wave for the verbal domain (e.g., Ruchkin et al., 1990, 1992, 1997a; Kusak et al., 2000; Kiss et al., 2007) and the (right) posterior negative slow wave for the visuospatial domain (e.g., Ruchkin et al., 1992, 1995, 1997b; Geffen et al., 1997; Löw et al., 1999; Bledowski et al., 2006; Pinal et al., 2014). Accordingly, in the single-task condition, we expected the (left) anterior negativity for the verbal domain compared with the visuospatial domain and the (right) posterior negativity for the visuospatial domain compared with the verbal domain.

In the dual-task condition, we expected that the concurrent motor task would interfere with WM also at the neurophysiological level, particularly with the visuospatial domain. Previous ERP studies have suggested two major slow waves for the manual actions: The posterior negative slow wave reflecting planning and execution of the action, and the anterior negative slow wave reflecting higher cognitive operations such as planning sequences and supporting final movement goals during sequential actions (e.g., van Schie and Bekkering, 2007; Bozzacchi et al., 2012; Westerholz et al., 2013, 2014; for a mini review, see Koester et al., 2016). Considering the previously suggested slow waves for the WM and manual actions in isolation, we would expect that the interactions of WM and grasping-and-placing movement would be visible at anterior and posterior scalp regions. Specifically, we expected that visuospatial ERPs in the dual-task would demonstrate different patterns than the ERPs in the single-task, i.e., domain-specific interference at the neurophysiological level. Moreover, given distinct cognitive operations of information in each WM process, we further expected that each process would share the cognitive resources with grasping-and-placing movement in a process-specific manner. Consequently, the memory costs would be non-identical for each WM process.

## Materials and Methods

### Participants

Thirty right-handed participants from students of Bielefeld University participated in the study. Due to the behavioral performance and EEG data quality, for the behavioral analyses 29 participants (21 females, 8 males, *M* age = 25 years, *SD* = 4.1), for the encoding process analyses 23 participants (20 females, 3 males, *M* age = 24.5 years, *SD* = 4), and for the retrieval process analyses 21 participants (18 females, 3 males, *M* age = 24.5 years, *SD* = 4.2) were entered into analyses.

All participants had normal or correct-to-normal vision and no known neurological disorder. Participants provided informed written consent and were compensated with either 15€ or 2-h of participation credits. This study was conducted in accordance with the ethical standards of the sixth revision of the Declaration of Helsinki and approved by the ethics committee at Bielefeld University.

### Materials

The stimulus events for the experimental task were presented on a 17-in flat-screen monitor with integrated speakers and a resolution of 1024 pixel × 768 pixel.

The stimuli for the verbal WM task were eighty pseudo-randomly chosen letter sequences, each consisting of eight consonants of the Latin alphabet (each consonant was 2 cm in height and width). Neither any abbreviation nor alphabetic order among the consonants was allowed. In addition, frequency of the presentation of each consonant was controlled. Each letter sequence was presented along a vertical axis centered at the middle of a monitor screen to avoid any possible visual field effect. The stimuli for the visuospatial WM task were eighty 4 × 4 symbol matrices. Each matrix consisted of a variation of eight symbols which were selected from three symbol types, i.e., triangle, circle, square, (each symbol was 2 cm in height and width). The symbols were placed at any random eight of sixteen equiprobable positions of the matrix which was presented at the center of a monitor screen.

**FIGURE 1:**
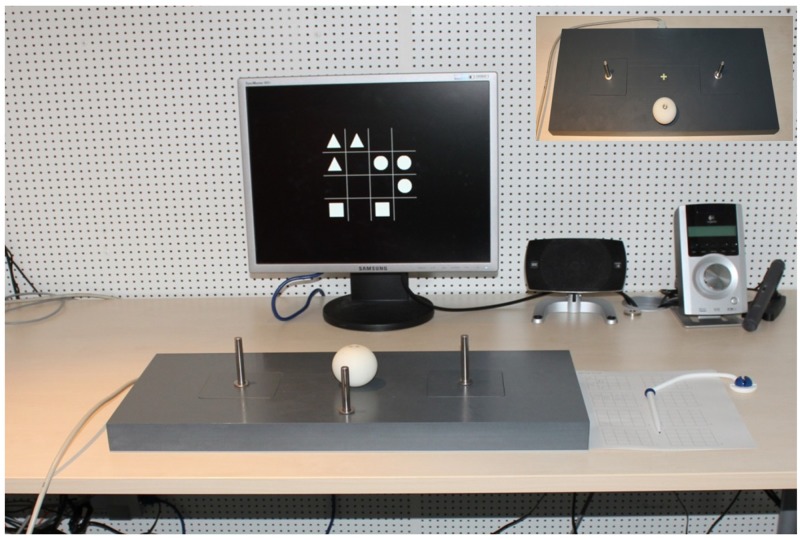
The experimental setup and task board for the manual-motor task (top view). Task board included two sticks (motor targets) and a sphere which is placed onto a start position as shown in the picture. The center of the task board is marked by a yellow cross.

A task board (4 cm × 60 cm × 28 cm) was used for the manual-motor task. The board consisted of a start position and two sticks (10 cm in height, 0.5 cm in width) as motor targets which required high precision movement. The motor targets were mounted on the left and right side of the board, being 15 cm away from the center which was marked by a yellow cross (**Figure 1**). The manual task required a sphere, 6 cm in diameter and furnished with a hole of 10 mm, to be fitted onto one of the sticks. The start position and motor targets were equipped with pressure sensitive micro switches which allowed for self-paced trial beginnings and ends.

### Design and Procedure

The study employed a 2 × 2 within-subject design with the factors task block (single block and dual block) and WM domain (verbal domain and visuospatial domain). Each experimental condition (i.e., single block verbal domain, single block visuospatial domain, dual block verbal domain and dual block visuospatial domain) consisted of 40 trials, resulting in a total of 160 experimental trials. Different WM stimulus sets were used in single and dual blocks to avoid repetition effect. The order of WM stimuli in each experimental condition was randomized.

There were four different versions of the experimental condition order (**Table 1**). That is, the experiment could start with either the single block or dual block, and each block could start with either the verbal task or visuospatial task. Participants first performed two WM tasks within the first block, and then started with the second block. We created four lists of a block sequence based on the possible orders of experimental conditions, which were also used for dividing the participants into four groups. Participants were randomly assigned to each group.

**Table 1:** Four groups of participants receiving different versions of the block sequence.

	First Block		Second Block	
		
	WM Task 1	WM Task 2	WM Task 1	WM Task 2
Group A	Single Block Visuospatial Task	Single Block Verbal Task	Dual Block Visuospatial Task	Dual Block Verbal Task
Group B	Single Block Verbal Task	Single Block Visuospatial Task	Dual Block Verbal Task	Dual Block Visuospatial Task
Group C	Dual Block Visuospatial Task	Dual Block Verbal Task	Single Block Visuospatial Task	Single Block Verbal Task
Group D	Dual Block Verbal Task	Dual Block Visuospatial Task	Single Block Verbal Task	Single Block Visuospatial Task


*Table shows the four groups of participants receiving different versions of a block sequence. Four lists of the block sequence were created based on the possible orders of experimental conditions.*

After giving written informed consent, participants were seated comfortably in an electrically shielded cabin where the experiment took place (**Figure 1**). Participants received instructions for the experimental task which also required maintaining stable posture and not blinking while executing the task. Single block required participants to perform either verbal or visuospatial version of the WM task. Dual block required participants to perform the WM task being embedded in the motor task. Single and dual blocks had the same fixed sequence of stimulus events which were initiated and terminated by participants themselves.

**FIGURE 2:**
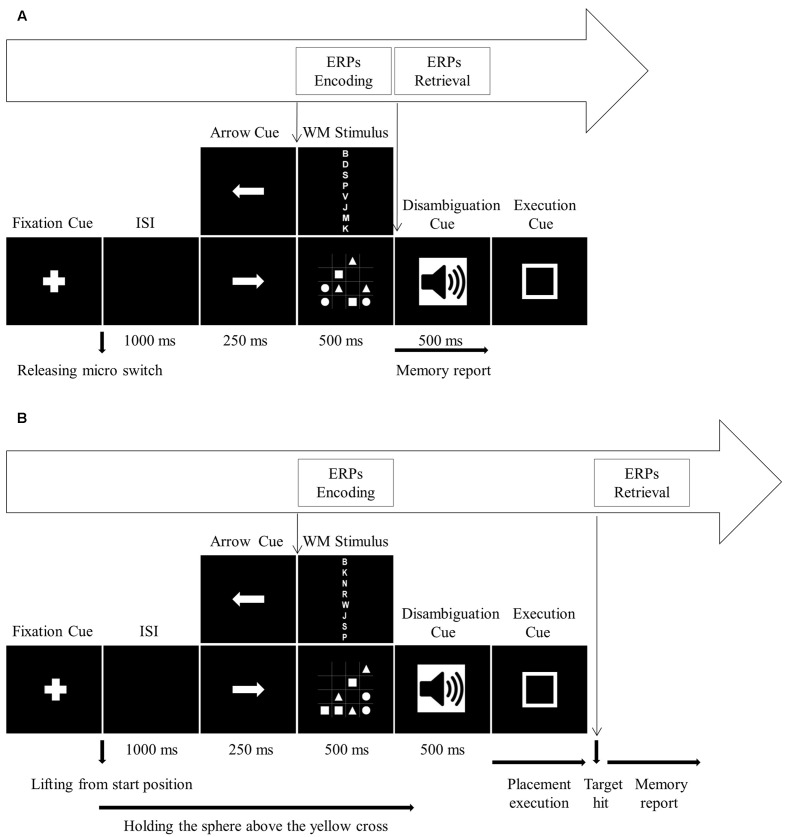
Timing of the stimulus events for **(A)** single block and **(B)** dual block. **(A)** For the single block, onset of WM stimulus is the reference event for encoding process ERPs, and onset of disambiguation cue is the reference event for the retrieval process ERPs. **(B)** For the dual block, onset of WM stimulus is the reference event for encoding process ERPs, and target hit is the reference event for the retrieval process ERPs.

In the single block, participants initiated the fixed sequence of stimulus events by pressing down on the micro switch mounted on the start position. First, a fixation cross appeared at the center of the monitor screen. When participants released the micro switch, the fixation cross disappeared. After an inter-stimulus interval (ISI) of 1000 ms, a directional arrow cue was presented for 250 ms. Following the arrow cue, a WM stimulus, either a letter sequence or a 4 × 4 matrix, was presented for 500 ms. During WM stimulus presentation, participants should encode the items into WM. The WM stimulus was followed by the disambiguation cue (500 ms duration), either a 400 or a 750 Hz tone, and movement execution cue. Since there was no motor task, participants ignored the arrow cue, disambiguation cue and movement execution cue, and reported WM items directly following the offset of WM stimulus (**Figure 2A**). After completing the WM report, participants self-initiated the next trial.

In the dual block, verbal and visuospatial versions of the WM task were combined with the motor task which required grasping the sphere, holding it and finally placing it on the motor target. The fixed sequence of stimulus events was identical to the sequence in the single block (**Figure 2B**). Initially, the sphere was at the start position. Participants grasped the sphere and pressed it down on the start position to initiate the stimulus events. First, a fixation cross appeared at the center of the monitor screen. Lifting the sphere from the start position started a 1000 ms ISI, during which participants transported the sphere to the center of the task board (yellow cross). Participants held the sphere above the yellow cross until the onset of movement execution cue. While participants were holding the sphere above the yellow cross, they were first presented with the directional arrow cue (250 ms duration) pointing to the left or right for indicating the motor target. The placement movement was planned according to this cue. Following the arrow cue, WM stimulus (500 ms duration), again either a letter sequence or a 4 × 4 matrix, was presented. Then, one of the disambiguation cues (500 ms duration) was presented. In the present study, participants ignored the disambiguation cue since it was only used to assure comparability with the study by Spiegel et al. (2013). After the disambiguation cue, the movement execution cue was visually presented. Participants started the placement movement with the onset of execution cue. The placement movement was performed by transporting the sphere from the yellow cross to the directed motor target and fitting the sphere onto the stick, which was also accepted as the termination of the manual task. After the manual task, participants reported the WM items. Then, participants placed the sphere back on the start position for the next trial. Participants were required to memorize as many letters or symbols as possible and move the sphere as quickly as possible but at a comfortable speed.

Both the verbal and visuospatial tasks required written report on the answers sheets provided. The verbal task required to memorize as many letters as possible and retrieve them independently of the serial order (i.e., only the identity). The answer sheet consisted of rectangle blank boxes with a left to right orientation. The visuospatial task required to memorize as many symbols as possible and retrieve the correct symbols in the correct positions within the matrix (i.e., identity and position). The answer sheet consisted of blank 4 × 4 matrices.

Prior to experimental blocks, participants completed 10 trials of each experimental condition for familiarization. Data from training blocks were not included into analyses. The stimulus presentation, response registration and timing were controlled by Presentation software (Neurobehavioral Systems, Albany, CA, United States). The entire experimental session lasted approximately 2 h.

### EEG Recordings

Electroencephalogram was recorded by a 64-channel amplifier (ANT)^2^. Ag/AgCL electrodes were arranged according to the international 10–10 system (Oostenveld and Praamstra, 2001) using WaveGuard EEG cap. Ocular artifacts were detected by four electrodes placed above and below the right eye and lateral to both eyes. Data were average-referenced during recording. The EEG was band-pass filtered (DC-138 Hz) and digitized at 512 Hz. The impedance of all electrodes was kept below 5 kV.

### Data Analysis

Regarding the behavioral data, the dependent variables were memory performance and execution time (ET). Memory performance for the verbal task was defined as the number of correctly reported letters independently of the serial order in the letter sequence. For the visuospatial task, it was defined as the number of correctly reported symbols in the correct position within the matrix. ET was defined as the time from the onset of disambiguation cue to the target hit only for the dual block.

Trials with placement errors were excluded from the memory performance, ET and EEG analyses. In addition, trials deviating more than 2.5 SD from the individual mean ET were excluded from ET analysis. For the memory performance analysis, a two-way repeated measure of analysis of variance (ANOVA) including the factors task block and WM domain was conducted on the arcsine transformed proportions of correct answers. For the ET analysis, a paired sample *t*-test was conducted.

Regarding the EEG data, given a lack of studies on the functional interactions of WM and manual actions mainly a data-driven approach seemed appropriate. EEG data from the verbal and visuospatial tasks both in single and dual blocks were analyzed separately for encoding and retrieval processes. First, EEG data were band-pass filtered from 0.1 to 30 Hz and re-referenced to the average mastoid electrodes. Then, stimulus-locked epochs for encoding and retrieval processes were extracted based on separate reference events (**Figure 2**). The encoding process epochs both in single and dual blocks were extracted time-locked to the WM stimulus onset with a 100 ms pre-stimulus baseline. These epochs included the time interval over the period of WM stimulus presentation (500 ms duration). The retrieval process epochs were extracted time-locked to the different reference events in single and dual blocks. To provide comparability with the study by Spiegel et al. (2013), we asked participants to report WM stimuli directly after the stimulus presentation in the single block, but after fitting the sphere onto the stick, i.e., the target hit, in the dual block. Therefore, the retrieval process epoch in the single block was extracted time-locked to the disambiguation cue onset with a 100 ms pre-stimulus baseline. Different from the single block epoch, the dual block epoch was extracted time-locked to the target hit with a 100 ms pre-stimulus baseline. Bearing in mind that movement artifacts may arise during longer time intervals, we kept the time interval for the retrieval process analyses shorter than the actual time required to complete WM retrieval. Therefore, the retrieval process epochs covered a duration of 1500 ms after the reference events. Given that participants could start reporting the WM stimuli directly after the presentation in the single block, we assumed that participants did not get into maintenance process, which is required for retaining the stimulus active when the perceptual input is not available (Jonides et al., 2008). The absence of maintenance process in the single block prevented us from comparing single and dual blocks for the movement interference with the maintenance process of WM domains. Consequently, our analyses were restricted to encoding and retrieval processes.

Ocular correction was done using the correction procedure of Gratton et al. (1983). Artifact detection was done using a peak-to-peak moving window approach. Epochs containing peak-to-peak amplitudes above the threshold of ±50 μV within a 200 ms window were rejected. Time epochs were visually double-checked for artifacts. If necessary, single bad channels causing the rejection of any epoch were interpolated. Then, data of the participants losing more than 50% of epochs of each experimental condition were excluded from further analyses. Therefore, encoding and retrieval processes had different number of participants, but the participants were kept equal for single and dual block comparisons. For example, the encoding process analyses had the same 23 participants both for single and dual block analyses. Afterward, grand average ERPs were computed at all electrode sites separately for encoding and retrieval processes of the verbal and visuospatial tasks both in single and dual blocks.

We determined four region-of-interests (ROI) based on the previous ERP studies on WM (e.g., Kiss et al., 2007; Studer et al., 2010; Pinal et al., 2014) and manual actions (e.g., van Schie and Bekkering, 2007; Westerholz et al., 2014; Koester and Schack, 2016). These ROIs were systematically aligned across the scalp: Left-anterior (LA), right-anterior (RA), left-posterior (LP), right-posterior (RP), and each included six recording electrodes. Electrodes for the LA were Fp1, AF7, AF3, F5, F3, and F1. Electrodes for the RA were Fp2, AF8, AF4, F6, F4, and F2. Electrodes for the LP were P5, P3, P1, PO7, PO5, and PO3. Electrodes for the RP were P6, P4, P2, PO8, PO6, and PO4.

Since there were different reference events for the retrieval process in single and dual blocks (for time-locking the ERPs), we could only compare the blocks qualitatively to examine the dual-task costs for verbal and visuospatial domains. Moreover, for the consistency of encoding and retrieval processes, we aimed for the qualitative comparison of single and dual blocks also for the encoding process. Therefore, we conducted three-way repeated measures of ANOVAs for each process separately in single and dual blocks (four ANOVAs in total). These ANOVAs included the factors WM domain (verbal and visuospatial), hemisphere (left and right) and anterior-posterior orientation of ROI (AP; anterior, posterior). The time intervals for statistical analyses were chosen as a combination of available ERP studies on WM and visual inspection of the grand average ERPs (e.g., Ruchkin et al., 1992; Bledowski et al., 2006; Pinal et al., 2014). The encoding process analyses included the time interval between 200 and 400 ms, and retrieval process analyses included two time intervals, i.e., early (250–650 ms) and late (800–1500 ms), both in single and dual blocks. By comparing the ERPs of verbal and visuospatial tasks in single and dual blocks separately, we qualitatively investigated if the ERP patterns would change in the presence of the motor task.

## Results

### Behavioral Results

Participants executed the grasping-and-placing movement correctly in 99% of trials of the verbal and visuospatial WM tasks in the dual block. Regarding the memory performance, the verbal task yielded on average 4.08 (*SD* = 0.36) correct letters in the single block and 3.98 (*SD* = 0.40) correct letters in the dual block. The visuospatial task yielded on average 3.73 (*SD* = 0.69) correct symbols in the single block and 3.07 (*SD* = 0.66) correct symbols in the dual block.

**FIGURE 3:**
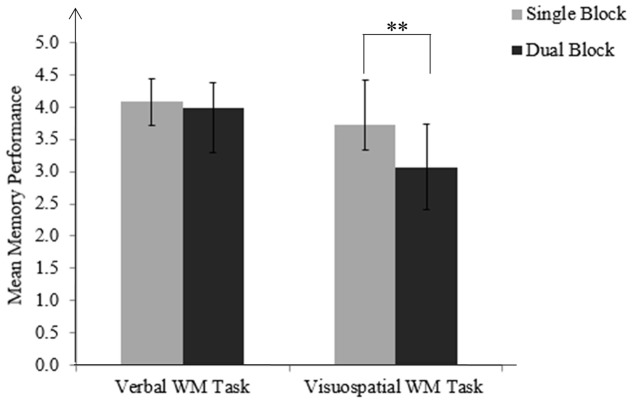
Mean memory performance for verbal and visuospatial WM tasks in single and dual blocks. Memory performance for the visuospatial task in the dual block is lower than the performance in the single block, i.e., domain-specific memory costs. Standard deviations are represented in the figure by the error bars attached to each column. ^∗∗^*p* < 0.001.

The two-way (Task block × WM domain) ANOVA revealed main effects of the block [*F*(1,28) = 55.38, *p* < 0.001, $\eta_{p}^{2}$ = 0.664), domain [*F*(1,28) = 23.62, *p* < 0.001, $\eta_{p}^{2}$ = 0.458], and a significant interaction [*F*(1,28) = 42.84, *p* < 0.001, $\eta_{p}^{2}$ = 0.605]. Follow-up paired sample *t*-tests indicated that for the visuospatial task, memory performance in the dual block was lower than performance in the single block, *t*(28) = 8.32, *p* < 0.001. For the verbal task, the difference between performances in single and dual blocks was not statistically significant, *t*(28) = 1.76, *p* = 0.089 (**Figure 3**).

Regarding ET in the dual block, there was no difference between the verbal task (*M* = 2177.6 ms, *SD* = 536) and visuospatial task (*M* = 2153.9 ms, *SD* = 438), *t*(28) = 0.24, *p* = 0.813.

### ERP Analyses Results

#### Encoding Process Analyses

**FIGURE 4:**
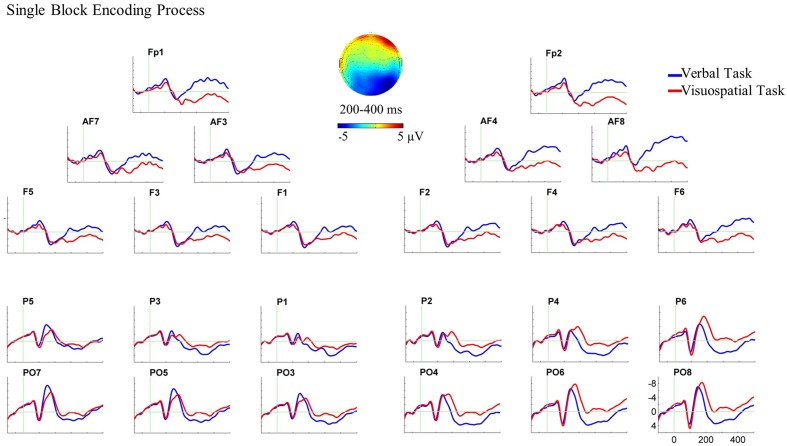
Grand average ERPs and the scalp map for the encoding process in the single block. ERPs are superimposed for the verbal task (blue line) and visuospatial task (red line) in this analysis and dual block analysis. In this and all subsequent ERP plots, six electrodes from each ROI are shown and are arrayed from left to right and from anterior to posterior as they were positioned on the scalp. Moreover, negativity is plotted upward in all ERP plots in the present study. In this ERP plot, stimulus onset occurred at 0 ms which was the onset of WM stimulus in the single block. The encoding process analysis in the single block showed the ERP effect in the bilateral anterior ROIs and in the right posterior ROI. The scalp map, which is plotted by subtracting the ERPs of the verbal task from the visuospatial task, represents the spatial scalp distribution of the ERP effect between 200 and 400 ms.

For the single block, the three-way ANOVA (WM domain × Hemisphere × AP) revealed a three-way interaction of domain, hemisphere and AP between 200 and 400 ms, *F*(1,22) = 17.74, *p* < 0.001, $\eta_{p}^{2}$ = 0.446.^3^ Following this interaction, paired sample *t*-tests were performed for each ROI. The verbal task elicited larger anterior negative slow waves compared with the visuospatial task over both hemispheres, [*t*(22) = -2.80, *p* = 0.011 for left and *t*(22) = -4.24, *p* < 0.001 for right]. In addition, the visuospatial task elicited a larger posterior negative slow wave compared with the verbal task over the right hemisphere, *t*(22) = 3.28, *p* = 0.003, (see **Figure 4** for the ERP plot and scalp map).

**FIGURE 5:**
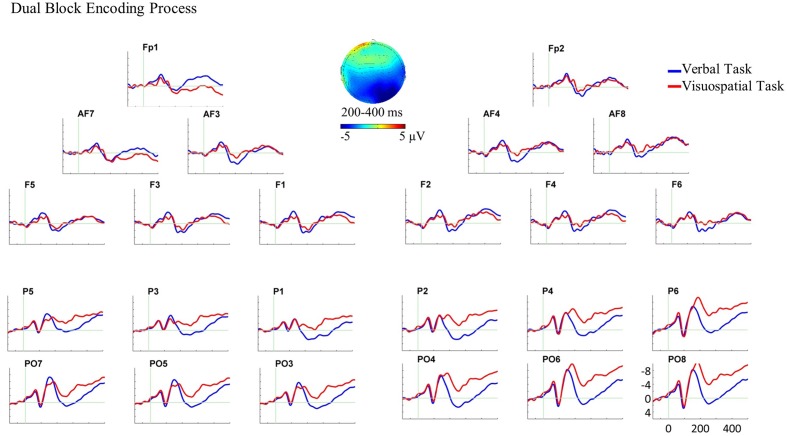
Grand average ERPs and the scalp map for the encoding process in the dual block. In this ERP plot, stimulus onset occurred at 0 ms which was the onset of WM stimulus in the dual block. The encoding process analysis in the dual block showed the ERP effect in the bilateral posterior ROIs. The scalp map, which is plotted by subtracting the ERPs of the verbal task from the visuospatial task, represents the spatial scalp distribution of the ERP effect between 200 and 400 ms.

For the dual block, the three-way ANOVA revealed a two-way interaction of domain and AP between 200 and 400 ms, *F*(1,22) = 11.24, *p* = 0.003, $\eta_{p}^{2}$ = 0.338. Subsequent paired sample *t*-tests were performed for anterior and posterior ROIs separately. The *t*-tests showed that the visuospatial task elicited larger posterior negative slow waves compared with the verbal task over both hemispheres, *t*(22) = 2.40, *p* = 0.025, (see **Figure 5** for the ERP plot and scalp map).

#### Retrieval Process Analyses

**FIGURE 6:**
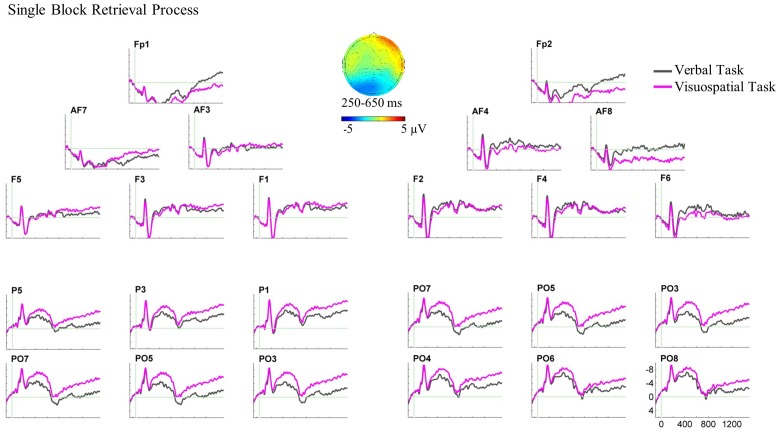
Grand average ERPs and the scalp map for the retrieval process ERPs in the single block. ERPs are superimposed for the verbal task (pink line) and visuospatial task (gray line) in this analysis and dual block analysis. In this ERP plot, stimulus onset occurred at 0 ms which was the onset of disambiguation cue in the single block. The retrieval process analysis in the single block showed the ERP effect in the bilateral posterior ROIs. The scalp map, which is plotted by subtracting the ERPs of the verbal task from the visuospatial task, represents the spatial scalp distribution of the ERP effect between 250 and 650 ms.

For the single block, the three-way ANOVA revealed a two-way interaction of domain × AP between 250 and 650 ms, *F*(1,20) = 12.40, *p* = 0.002, $\eta_{p}^{2}$ = 0.383. Two paired sample *t*-tests were performed for anterior and posterior ROIs separately. Results of the *t*-tests showed larger posterior negative slow waves for the visuospatial task compared with the verbal task over both hemispheres, *t*(20) = 2.19, *p* = 0.041 (see **Figure 6** for the ERP plot and scalp map).

Further three-way ANOVA in the later time window, 800–1500 ms, also revealed a two-way interaction of domain × AP, *F*(1,20) = 9.124, *p* = 0.007, $\eta_{p}^{2}$ = 0.313. Although the ERPs and scalp distribution visually showed similar differences between the WM tasks in this time window, the *t*-tests showed differences neither for anterior nor for posterior ROIs.

**FIGURE 7:**
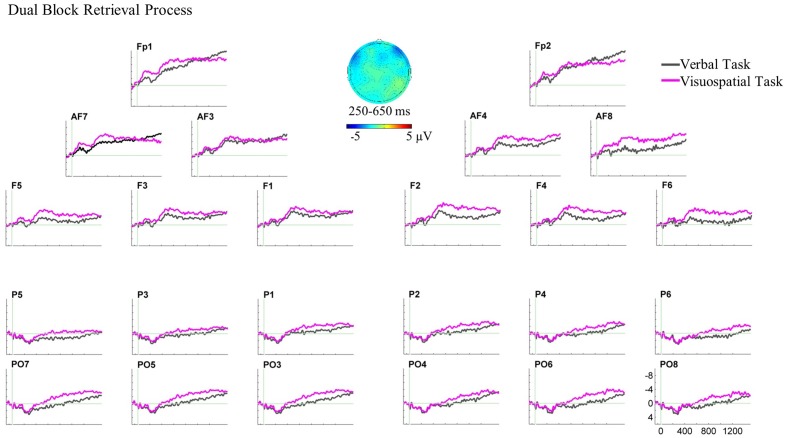
Grand average ERPs and the scalp map for the retrieval process ERPs in the dual block. In this ERP plot, stimulus onset occurred at 0 ms which was the target hit in the dual block. The retrieval process analysis in the dual block showed no significant ERP effect.

For the dual-task block, three-way ANOVAs showed neither a main effect nor interaction of the factors in any time window (see **Figure 7** for the ERP plot and scalp map).^4^^,^^5^

## Discussion

Here, we investigated the neurophysiological correlates of the WM and manual action interactions. More specifically, we investigated whether the interference of a motor task (including a prepared grasping-and-placing movement) pertains to the encoding and retrieval processes of verbal and visuospatial domains. The behavioral analyses, replicating the behavioral study by Spiegel et al. (2013), showed domain-specific memory costs for the visuospatial domain. The ERP analyses showed the domain-specific interference also at the neurophysiological level, which is further process-specific to the encoding. In support of our hypotheses, we provide an initial neurophysiological evidence for the domain- and process-specific manual action interactions with WM (with the visuospatial domain during encoding process).

### Behavioral Memory Costs

In the single block, memory performances for verbal and visuospatial tasks were on average 3–4 items, which are consistent with the proposed limited capacity of WM (e.g., Cowan, 2000). Importantly, performing additional prepared movement decreased memory performance for the visuospatial task. There was no marked memory performance decrease for the verbal task when comparing the single and dual blocks. That is, memory costs of the motor task seems to be specific to the visuospatial domain. On the one hand, this domain specificity is in contrast with the ‘basic concurrence cost’ hypothesis which views the dual-task costs as general costs (Logan and Fischman, 2011). According to this hypothesis, memory performance should also decrease for the verbal task. On the other hand, our findings are in line with the findings of behavioral study by Spiegel et al. (2013), which also showed larger memory costs for the visuospatial domain compared with the verbal domain.

The selective interference of the motor task with the visuospatial domain is consistent with the multi-component model of WM positing separate verbal and visuospatial domains which draw on specialized cognitive resources (Baddeley, 2000). According to this model, both domains consist of a passive capacity-limited perceptual store and an active rehearsal mechanism which prevents material-specific information from decaying. For the verbal domain the covert articulatory rehearsal mechanisms, and for the visuospatial domain the motor processes have been initially suggested to serve to hold information in WM (e.g., Baddeley, 1992; Belopolsky and Theeuwes, 2009; Logie, 2011). Consistently, when performed concurrently, many forms of motor actions have been shown to produce interference with the visuospatial domain. These include finger tapping (e.g., Smyth et al., 1988), pointing (e.g., Hale et al., 1996), eye movements (e.g., Lawrence et al., 2001), arm movements (e.g., Quinn and Ralston, 1986; Lawrence et al., 2001) and complex movements (e.g., Cortese and Rossi-Arnaud, 2010). Recently, attention has been also suggested as an alternative cognitive resource for serving to hold information in the visuospatial domain (e.g., Postle et al., 2004; Godijn and Theeuwes, 2012). In the present experiment, performing the grasping-and-placing movement required participants to shift attention not only for holding the sphere centrally while encoding WM items, but also for placing the sphere onto the motor target. Moreover, placing the sphere precisely onto the target required oculomotor control (Spiegel et al., 2013). Therefore, it is suggested that attention and oculomotor control are the resources which are required by both visuospatial task and motor task. The sharing of limited attentional and oculomotor resources might provide a potential mechanism to explain domain-specific memory costs in our data.

An alternative explanation for the domain-specific memory costs might be that the visuospatial character of the arrow cue, not the motor task itself, interfered selectively with the visuospatial memory. In the present dual-task paradigm, participants were presented with a directional arrow cue pointing one of the motor targets. Depending on this cue, participants could plan the subsequent placement movement. As an alternative, it might be suggested that the direction of the arrow cue was kept in WM as an abstract (i.e., symbolically coded) representation and not as a planned, subsequent placement movement. Therefore, the abstract representation of the arrow cue required the same capacity-limited cognitive resources which were also required for keeping the visuospatial memory items in WM. These shared capacity-limited cognitive resources, thus, resulted in the memory costs for the visuospatial task. However, we do not consider this as the main explanation of the present domain-specific memory costs for the following reasons. A recent behavioral study by Spiegel et al. (2013), which used the same experimental setup and procedure, have shown that participants indeed used the arrow cue for planning the placement movement. Spiegel et al. (2013) investigated the interference of movement (re-)planning with verbal and visuospatial WM domains. As in our study, participants were presented with an arrow cue depending on which they performed the placement movement toward either the left or right motor target. After the arrow cue, a WM stimulus was presented. Then, an auditory cue (either a high or low tone) was presented. Depending on the tone, participants placed the sphere either on the motor target pointed by the arrow cue (keeping the initial movement) or on the other motor target (changing the initial movement for 20% of trials). The results revealed that changing the movement plan (re-planning) decreased the memory performance for both verbal and visuospatial tasks.

Now, if the arrow cue had been represented just as an abstract symbol, then one would expect that participants must, upon the auditory cue, access the symbolic code, compute a motor target position and execute the movement. Consequently, no re-planning effect should be observed (as there would not have been any planning in the first place). However, such a re-planning effect was consistently found by Spiegel et al. (2012, 2013). Therefore, we argue that the arrow cue was not represented as a symbol that interfered with the visuospatial memory. Rather, it is suggested that the arrow cue was used for motor planning and the prepared movement itself interfered with the visuospatial memory. Future research may confirm this suggestion by using, for example, different non-spatial cues for movement planning.

### Encoding Process and ERPs

In the single block, ERPs for the verbal and visuospatial tasks started to diverge at bilateral anterior and right posterior recording sites about 200 ms following the onset of stimulus presentation (**Figure 4**). ERP differences observed in this time interval showed larger bilateral anterior negativities for the encoding of verbal material compared with visuospatial material, and a larger right posterior negativity for the encoding of visuospatial material compared with verbal material. The different scalp distribution suggests, consistent with our hypotheses, that the encoding of verbal and visuospatial material are different cognitive processes and seem to arise from non-identical neural sources. These findings support previous ERP studies that have also shown different ERPs for the encoding of verbal and visuospatial material (e.g., Ruchkin et al., 1990, 1992; Bosch et al., 2001).

This right posterior negativity has been suggested to reflect the perception and identification of visuospatial material for encoding it into visuospatial store (e.g., Ruchkin et al., 1995, 1997b; Mecklinger, 1998). Previously, the anterior negativity, particularly left hemisphere dominant, has been suggested to reflect the articulatory rehearsal mechanisms for the maintenance of verbal material (e.g., Ruchkin et al., 1990, 1992, 1997a). The present study shows that an anterior negativity can also be elicited for the encoding of verbal material. This anterior negativity might reflect that participant started rehearsing the letters while stimuli were still present. Obviously, rehearsing may last longer than the actual analyzed epoch for the encoding. Therefore, this interpretation should be approached with caution.

In the dual block, ERP analyses showed a differential cortical activity for the WM tasks at bilateral posterior recording sites between 200 and 400 ms (**Figure 5**). This bilateral posterior ERP difference reflects the larger negativities for the encoding of visuospatial material compared with verbal material. There was no significant ERP difference between the tasks at anterior recording sites.

Comparing the single and dual blocks in terms of the patterns of ERP differences between the verbal and visuospatial tasks, we suggest that the posterior effect was not the same in single and dual blocks. Whereas the encoding of visuospatial material compared with the verbal material elicited a larger right posterior negativity in the single block, it elicited larger bilateral posterior negativities in the dual block. These findings fit to the reports in the literature that have shown either the right dominant or bilaterally distributed posterior negativity for the encoding of visuospatial material (e.g., Ruchkin et al., 1995, 1997b; Mecklinger, 1998). In line with these studies, we argue that the present posterior negativity reflects the encoding of visuospatial material into WM, and that additional motor task changes the neuro-cognitive processes underlying this operation.

Regarding the anterior null effect in the dual block, on the one hand, it is highly unlikely that there was no or a reduced involvement of the verbal domain since the high memory performance in the dual block showed that verbal material was still encoded. On the other hand, the visuospatial task, at least qualitatively, seems to elicit increased anterior negativity in the dual block compared with the single block (**Figures 4**, **5**). It can be assumed that the dual block was more complex and difficult than the single block. Therefore, it is possible that participants used, at least partly, verbalization strategies for the encoding of visuospatial material in the dual block. Another alternative could be that the more difficult (dual) task demanded more attentional resources. Previous ERP studies have shown that high cognitive (attentional) processing which is required for the encoding of visuospatial material in the presence of an increased WM load results in an increased anterior negativity (e.g., Ruchkin et al., 1995; Awh et al., 2000; Studer et al., 2010; Luu et al., 2014). Although we cannot decide between these interpretations with the present data, in either case the encoding of visuospatial material in our study should elicit an increased anterior negativity in the dual block compared with the single block. Consequently, the comparison of the verbal and the visuospatial ERPs at anterior recording sites may not result in a statistically significant amplitude difference. Hence, an increased task difficulty might explain the non-significant ERP effect at anterior recording sites. Further research is needed to confirm this suggestion.

These findings suggest that the ERP differences between the verbal and visuospatial tasks qualitatively change from single block to dual block in terms of the scalp topography. We interpret this qualitative change as reflecting the changes in the neuro-cognitive processes underlying the encoding of visuospatial material in the dual block (both at anterior and posterior recording sites). Hereby, these findings reflect the neurophysiological memory costs of motor task for the encoding process of visuospatial domain.

### Retrieval Process and ERPs

In the single block, ERPs for the verbal and the visuospatial tasks started to diverge at bilateral posterior recording sites about 250 ms following the sphere placement onto the stick and continued until 650 ms (**Figure 6**). This bilateral posterior difference shows larger negativities for the visuospatial task compared with the verbal task, and it is supposed to reflect the retrieval of visuospatial material. There was no significant ERP difference between the tasks at anterior recording sites.

The anterior null effect might suggest that the retrieval of verbal and visuospatial material is equally difficult, given that participants could retrieve verbal and visuospatial material. If this were so, we should not find any ERP difference for the retrieval process, but there was bilateral posterior effect reflecting the retrieval of visuospatial material. We expected such a posterior effect which confirms our hypothesis. This effect is also in line with reports in the literature (e.g., Bledowski et al., 2006; Pinal et al., 2014), which have shown posterior negativities for the retrieval of visuospatial material. Moreover, this posterior retrieval effect in the present study demonstrates that reliable ERPs can be obtained during overt movement execution in a complex experimental setting. Regarding the anterior null effect, we cannot fundamentally rule out that the retrieval process involves neural generators that are difficult to trace, and thus we obtained null effect at anterior recording sites. Conceivably, frontal motor-related cortical activity may conceal retrieval effect (e.g., Westerholz et al., 2013, 2014). Future research is necessary to investigate this interpretation.

In the dual block, there was an ERP difference between the verbal and visuospatial tasks neither at anterior nor posterior recording sites (**Figure 7**). First, we note that the retrieval ERPs in the dual block did not show early components (N1/P2) which were present for the retrieval ERPs in the single block. Instead, the dual block retrieval ERPs showed steady, more or less constant amplitude both at anterior and posterior recording sites. For the anterior recording sites, the null ERP effect in the dual block fits to the anterior null effect in the single block. In contrast, the bilateral posterior effect in the single block was not found in the dual block. Although, it is not fully understood yet, the persistent ERPs may be potentially a hint for ongoing cognitive activity that would be expected for retrieval processes. Conceivably, if there was a persistent amplitude difference between the verbal and visuospatial ERPs that was also present during the baseline period, one would artificially eliminate a constant ERP amplitude difference. Unfortunately, there is no preceding fixed duration event for time-locking that would permit a more appropriate retrieval ERP analysis in the dual block.

For the claim that the retrieval process is affected by the concurrent motor task, one would need to show changes in the ERP patterns between single and dual blocks. There was an anterior effect neither in the single block nor in the dual block. That is, the ERP patterns at the anterior recording sites did not change. In contrast, in the single block, we observed the bilateral posterior effect reflecting the retrieval of visuospatial material, which was not observed in the dual block. Although, this seems to be a change in the ERP patterns, it is also conceivable that absence of a posterior effect in the dual block was due to methodological circumstances (e.g., a constant effect in the baseline) and not the absence of the difference between the retrieval processes of verbal and visuospatial domains. Moreover, analyzing the retrieval processes in single and dual blocks based on different reference events might have made it difficult to show the changes in ERP patterns in the presence of the motor task. Therefore, we do not consider the present evidence sufficient for strictly concluding that the ERP patterns changed between single and dual blocks. Further research is needed to characterize the functional interaction between the retrieval process and manual actions.

## Conclusion

The present study provides evidence to extend our understanding of the cognitive mechanisms of motor action control. We focused on the WM as one of the widely studied cognitive domains in relation to motor action control and manual actions (specifically, a manual-motor movement including grasping an object and placing it on a target position) as one of the cognitively demanding motor actions. More importantly, as our main objective, we focused on the interactions of WM and manual action, cortical neural activity and how we can evaluate these by means of ERPs.

First, we replicate the behavioral findings of the study by Spiegel et al. (2013) by showing the domain-specific memory costs of the prepared movement for the visuospatial domain. Second, as our main objective, we provide an initial neurophysiological characterization of the functional interactions of WM and manual action control. More specifically, our study examined the encoding and retrieval processes of verbal and visuospatial domains and whether these processes are affected by prepared movement. Our results have shown a difference between single and dual blocks for the encoding process of the visuospatial task. That is, our study has established neurophysiologically that at least the encoding of visuospatial material is affected by the concurrent motor task. This finding points toward the functional importance of the encoding process with regard to the motor interference with WM. Third, we report reliable ERPs in a complex experimental setting including overt movement execution, which extends the situations of mere spoken language (Koester and Schiller, 2008; Ganushchak et al., 2011) and mere grasping (van Schie and Bekkering, 2007; Westerholz et al., 2013, 2014).

Future investigations are required for characterizing the functional interactions between manual actions and both the retrieval and maintenance processes. Moreover, future quantitative statistical comparisons beyond the qualitative comparisons of the single and dual blocks are required. These investigations should provide a better understanding of the distinct spatio-temporal characteristics of neuro-cognitive resources shared by manual actions and separate WM processes, and functional interdependence of WM and motor action control. More generally, the present study points toward potential neurobiological underpinnings of motor action control and its interactions with WM.

## Author Contributions

Conceived and designed the study: RGC, TS, and DK. Performed data collection: RGC. Analyzed and interpreted data: RGC and DK. Contributed to the interpretation and preparation of the manuscript: RGC, TS, and DK.

## Conflict of Interest Statement

The authors declare that the research was conducted in the absence of any commercial or financial relationships that could be construed as a potential conflict of interest.

## Footnotes

1 Note that we did not examine ‘grasping’ *per se* in this study.

2 http://www.ant-neuro.com

3 We conducted another three-way ANOVA for the time interval between 200 and 500 ms considering that the encoding process may take longer. This ANOVA also revealed the same statistical pattern for the ERP effects as the 200–400 ms analysis.

4 As aforementioned, we think that qualitative comparisons between single and dual blocks are more appropriate compared to the quantitative comparisons because of the different task requirements in the current study. Nevertheless, we conducted two four-way repeated measures of ANOVAs separately for encoding and retrieval processes. These ANOVAs included the factors *task block, WM domain, hemisphere* and *AP*. For the encoding process, the ANOVA revealed a significant four-way interaction between 200 and 400 ms, *F*(1,22) = 4.96, *p* = 0.036, $\eta_{p}^{2}$ = 0.184. For the retrieval process, the ANOVA revealed a significant three-way interaction of *task block, WM domain* and *AP* between 250 and 650 ms, *F*(1,20) = 12.64, *p* = 0.002, $\eta_{p}^{2}$ = 0.387 with both interactions supporting qualitative difference among the present tasks.

5 See Supplementary Figures 1–6 for the mean ERP values entered in the three-way ANOVAs for encoding and retrieval process in single and dual blocks.

## References

[B1] Ansuini C. Giosa L. Turella L. Altoè G. Castiello U. (2008). An object for an action, the same object for other actions: effects on hand shaping. *Exp. Brain Res.* 85 111–119. 10.1007/s00221-007-1136-4 17909766

[B2] Awh E. Anllo-Vento L. Hillyard S. A. (2000). The role of spatial selective attention in working memory for locations: evidence from event-related potentials. *J. Cogn. Neurosci.* 12 840–847. 10.1162/089892900562444 11054925

[B3] Baddeley A. (1992). Working memory. *Science* 255 556–559. 10.1126/science.1736359 1736359

[B4] Baddeley A. (2000). The episodic buffer: a new component of working memory? *Trends Cogn. Sci.* 4 417–423. 11058819 10.1016/s1364-6613(00)01538-2

[B5] Baddeley A. (2003). Working memory: looking back and looking forward. *Nat. Rev. Neurosci.* 4 829–839. 10.1038/nrn1201 14523382

[B6] Baddeley A. D. Hitch G. J. (1974). “Working memory,” in *Recent Advances in Learning and Motivation*, ed. Bower G. A. (New York, NY: Academic Press), 47–89.

[B7] Belopolsky A. V. Theeuwes J. (2009). Inhibition of saccadic eye movements to locations in spatial working memory. *Atten. Percept. Psychophys.* 71 620–631. 10.3758/APP.71.3.620 19304651

[B8] Binsted G. Rolheiser T. M. Chua R. (2006). Decay in visuomotor representations during manual aiming. *J. Mot. Behav.* 38 82–87. 10.3200/JMBR.38.2.82-87 16531391

[B9] Bledowski C. Kadosh K. C. Wibral M. Rahm B. Bittner R. A. Hoechstetter K. (2006). Mental chronometry of working memory retrieval: a combined functional magnetic resonance imaging and event-related potentials approach. *J. Neurosci.* 26 821–829. 10.1523/JNEUROSCI.3542-05.2006 16421302 PMC6675353

[B10] Bosch V. Mecklinger A. Friederici A. D. (2001). Slow cortical potentials during retention of object, spatial, and verbal information. *Cogn. Brain Res.* 10 219–237. 10.1016/S0926-6410(00)00040-9 11167047

[B11] Bozzacchi C. Giusti M. A. Pitzalis S. Spinelli D. Di Russo F. (2012). Awareness affects motor planning for goal-oriented actions. *Biol. Psychol.* 89 503–514. 10.1016/j.biopsycho.2011.12.020 22234365

[B12] Castiello U. (2005). The neuroscience of grasping. *Nat. Rev. Neurosci.* 6 726–736. 10.1038/nrn1744 16100518

[B13] Castiello U. Begliomini C. (2008). The cortical control of visually guided grasping. *Neuroscientist* 14 157–170. 10.1177/1073858407312080 18219055

[B14] Cortese A. Rossi-Arnaud C. (2010). Working memory for ballet moves and spatial locations in professional ballet dancers. *Appl. Cogn. Psychol.* 24 266–286. 10.1002/acp.1593

[B15] Cowan N. (2000). The magical number 4 in short-term memory: a reconsideration of mental storage capacity. *Behav. Brain Sci.* 24 87–185. 10.1017/S0140525X01003922 11515286

[B16] Creem S. H. Proffitt D. R. (2001). Grasping objects by their handles: a necessary interaction between cognition and action. *J. Exp. Psychol.-Hum. Percept. Perform.* 27 218–228. 10.1037/0096-1523.27.1.218 11248935

[B17] Deubel H. Schneider W. X. Paprotta I. (1998). Selective dorsal and ventral processing: evidence for a common attentional mechanism in reaching and perception. *Vis. Cogn.* 5 81–107. 10.1080/713756776

[B18] Elliott D. Helsen W. F. Chua R. (2001). A century later: Woodworth’s (1899) two-component model of goal-directed aiming. *Psychol. Bull.* 127 342–357. 10.1037/0033-2909.127.3.342 11393300

[B19] Fiehler K. Bannert M. M. Bischoff M. Blecker C. Stark R. Vaitl D. (2011). Working memory maintenance of grasp-target information in the human posterior parietal cortex. *Neuroimage* 54 2401–2411. 10.1016/j.neuroimage.2010.09.080 20932912

[B20] Fournier L. R. Behmer L. P. Jr. Stubblefield A. M. (2014). Interference due to shared features between action plans is influenced by working memory span. *Psychon. Bull. Rev.* 21 1524–1529. 10.3758/s13423-014-0627-0 24715506

[B21] Friedman D. Johnson R. (2000). Event-related potential (ERP) studies of memory encoding and retrieval: a selective review. *Microsc. Res. Tech.* 51 6–28. 10.1002/1097-0029(20001001)51:1<6::AID-JEMT2>3.0.CO;2-R 11002349

[B22] Galletti C. Kutz D. F. Gamberini M. Breveglieri R. Fattori P. (2003). Role of the medial parieto-occipital cortex in the control of reaching and grasping movements. *Exp. Brain Res.* 153 158–170. 10.1007/s00221-003-1589-z 14517595

[B23] Ganushchak L. Y. Christoffels I. K. Schiller N. O. (2011). The use of electroencephalography in language production research: a review. *Front. Psychol.* 2:208 10.3389/fpsyg.2011.00208 PMC3164111 21909333

[B24] Geffen G. M. Wright M. J. Green H. J. Gillespie N. A. Smyth D. C. Evans D. M. (1997). Effects of memory load and distraction on performance and event-related slow potentials in a visuospatial working memory task. *J. Cogn. Neurosci.* 9 743–757. 10.1162/jocn.1997.9.6.743 23964597

[B25] Glover S. (2004). Planning and control in action. *Behav. Brain Sci.* 27 57–69. 10.1017/s0140525x04520022 15481943

[B26] Glover S. Rosenbaum D. A. Graham J. Dixon P. (2004). Grasping the meaning of words. *Exp. Brain Res.* 154 103–108. 10.1007/s00221-003-1659-2 14578997

[B27] Godijn R. Theeuwes J. (2012). Overt is no better than covert when rehearsing visuo-spatial information in working memory. *Mem. Cogn.* 40 52–61. 10.3758/s13421-011-0132-x PMC3246584 21769706

[B28] Grafton S. T. (2010). The cognitive neuroscience of prehension: recent developments. *Exp. Brain Res.* 204 475–491. 10.1007/s00221-010-2315-2 20532487 PMC2903689

[B29] Gratton G. Coles M. G. Donchin E. (1983). A new method for off-line removal of ocular artifact. *Electroen. Clin. Neurophysiol.* 55 468–484. 10.1016/0013-4694(83)90135-9 6187540

[B30] Grezes J. Tucker M. Armony J. Ellis R. Passingham R. E. (2003). Objects automatically potentiate action: an fMRI study of implicit processing. *Eur. J. Neurosci.* 17 2735–2740. 10.1046/j.1460-9568.2003.02695.x 12823480

[B31] Guillery E. Mouraux A. Thonnard J.-L. (2013). Cognitive-motor interference while grasping, lifting and holding objects. *PLoS ONE* 8:e80125 10.1371/journal.pone.0080125 PMC3820537 24244626

[B32] Hale S. Myerson J. Rhee S. H. Weiss C. S. Abrams R. A. (1996). Selective interference with the maintenance of location information in working memory. *Neuropsychology* 10 228 10.1016/j.neuropsychologia.2008.02.003

[B33] Herbort O. Butz M. V. (2010). Planning and control of hand orientation in grasping movements. *Exp. Brain Res.* 202 867–878. 10.1007/s00221-010-2191-9 20195848

[B34] Herbort O. Butz M. V. (2011). Habitual and goal-directed factors in (everyday) object handling. *Exp. Brain Res.* 213 371–382. 10.1007/s00221-011-2787-8 21748333

[B35] Hesse C. Franz V. H. (2010). Grasping remembered objects: exponential decay of the visual memory. *Vision Res.* 50 2642–2650. 10.1016/j.visres.2010.07.026 20692279

[B36] Hillyard S. A. Kutas M. (1983). Electrophysiology of cognitive processing. *Annu. Rev. Psychol.* 34 33–61. 10.1146/annurev.ps.34.020183.000341 6338812

[B37] Jonides J. Lewis R. L. Nee D. E. Lustig C. A. Berman M. G. Moore K. S. (2008). The mind and brain of short-term memory. *Annu. Rev. Psychol.* 59 193–224. 10.1146/annurev.psych.59.103006.093615 17854286 PMC3971378

[B38] Kiss I. Watter S. Heisz J. J. Shedden J. M. (2007). Control processes in verbal working memory: an event-related potential study. *Brain Res.* 1172 67–81. 10.1016/j.brainres.2007.06.083 17803980

[B39] Koester D. Schack T. (2016). Action priority: early neurophysiological interaction of conceptual and motor representations. *PLoS ONE* 11:e0165882 10.1371/journal.pone.0165882 PMC5156427 27973539

[B40] Koester D. Schack T. Westerholz J. (2016). Neurophysiology of grasping actions: evidence from ERPs. *Front. Psychol.* 7:1996 10.3389/fpsyg.2016.01996 PMC5177652 28066310

[B41] Koester D. Schiller N. O. (2008). Morphological priming in overt language production: electrophysiological evidence from Dutch. *Neuroimage* 42 1622–1630. 10.1016/j.neuroimage.2008.06.043 18674626

[B42] Kohler J. Isenberg C. Schönle P. W. Inbar G. F. Conrad B. (1989). The role of short-term visuo-spatial memory in control of rapid multi-joint prehensive movements. *Eur. Arch. Psychiatr. Neurol. Sci.* 238 189–195. 10.1007/BF00381463 2759151

[B43] Kusak G. Grune K. Hagendorf H. Metz A.-M. (2000). Updating of working memory in a running memory task: an event-related potential study. *Int. J. Psychophysiol.* 39 51–65. 10.1016/S0167-8760(00)00116-1 11120347

[B44] Lawrence B. M. Myerson J. Oonk H. M. Abrams R. A. (2001). The effects of eye and limb movements on working memory. *Memory* 9 433–444. 10.1080/09658210143000047 11594362

[B45] Lindemann O. Stenneken P. van Schie H. T. Bekkering H. (2006). Semantic activation in action planning. *J. Exp. Psychol.-Hum. Percept. Perform.* 32 633–643. 10.1037/0096-1523.32.3.633 16822129

[B46] Logan S. W. Fischman M. G. (2011). The relationship between end-state comfort effects and memory performance in serial and free recall. *Acta Psychol.* 137 292–299. 10.1016/j.actpsy.2011.03.009 21497330

[B47] Logie R. H. (2011). The functional organization and capacity limits of working memory. *Curr. Dir. Psychol. Sci.* 20 240–245. 10.1177/0963721411415340

[B48] Löw A. Rockstroh B. Cohen R. Hauk O. Berg P. Maier W. (1999). Determining working memory from ERP topography. *Brain Topogr.* 12 39–47. 10.1023/A:1022229623355 10582564

[B49] Luu P. Caggiano D. M. Geyer A. Lewis J. Cohn J. Tucker D. M. (2014). Time-course of cortical networks involved in working memory. *Front. Hum. Neurosci.* 8:4 10.3389/fnhum.2014.00004 PMC3905217 24523686

[B50] Manoach D. S. Greve D. N. Lindgren K. A. Dale A. M. (2003). Identifying regional activity associated with temporally separated components of working memory using event-related functional MRI. *Neuroimage* 20 1670–1684. 10.1016/j.neuroimage.2003.08.002 14642477

[B51] Mecklinger A. (1998). On the modularity of recognition memory for object form and spatial location: a topographic ERP analysis. *Neuropsychologia* 36 441–460. 10.1016/S0028-3932(97)00128-0 9699951

[B52] Mecklinger A. Gruenewald C. Besson M. Magnié M. N. von Cramon D. Y. (2002). Separable neuronal circuitries for manipulable and non-manipulable objects in working memory. *Cereb. Cortex* 12 1115–1123. 10.1093/cercor/12.11.1115 12379600

[B53] Mecklinger A. Gruenewald C. Weiskopf N. Doeller C. F. (2004). Motor affordance and its role for visual working memory: evidence from fMRI studies. *Exp. Psychol.* 51 258–269. 10.1027/1618-3169.51.4.258 15620227

[B54] Olivier E. Davare M. Andres M. Fadiga L. (2007). Precision grasping in humans: from motor control to cognition. *Curr. Opin. Neurobiol.* 17 644–648. 10.1016/j.conb.2008.01.008 18337084

[B55] Oostenveld R. Praamstra P. (2001). The five percent electrode system for high-resolution EEG and ERP measurements. *Clin. Neurophysiol.* 112 713–719. 10.1016/S1388-2457(00)00527-7 11275545

[B56] Pashler H. (1994). Dual-task interference in simple tasks: data and theory. *Psychol. Bull.* 116 220–244. 10.1037/0033-2909.116.2.220 7972591

[B57] Pinal D. Zurrón M. Díaz F. (2014). Effects of load and maintenance duration on the time course of information encoding and retrieval in working memory: from perceptual analysis to post-categorization processes. *Front. Hum. Neurosci.* 8:165 10.3389/fnhum.2014.00165 PMC3978287 24744715

[B58] Postle B. R. Awh E. Jonides J. Smith E. E. D’Esposito M. (2004). The where and how of attention-based rehearsal in spatial working memory. *Cogn. Brain Res.* 20 194–205. 10.1016/j.cogbrainres.2004.02.008 15183391

[B59] Postle B. R. Idzikowski C. Della Sala S. Logie R. H. Baddeley A. D. (2006). The selective disruption of spatial working memory by eye movements. *Q. J. Exp. Psychol.* 59 100–120. 10.1080/17470210500151410 PMC1414070 16556561

[B60] Quinn J. G. Ralston G. E. (1986). Movement and attention in visual working memory. *Q. J. Exp. Psychol.* 38 689–703. 10.1080/14640748608401621 3809576

[B61] Rosenbaum D. A. Chapman K. M. Weigelt M. Weiss D. J. van der Wel R. (2012). Cognition, action, and object manipulation. *Psychol. Bull.* 138 924–946. 10.1037/a0027839 22448912 PMC3389205

[B62] Rosenbaum D. A. Herbort O. van der Wel R. Weiss D. J. (2014). What’s in a Grasp? *Am. Sci.* 102 366–373. 10.1511/2014.110.366

[B63] Ruchkin D. S. Berndt R. S. Johnson R. Ritter W. Grafman J. Canoune H. L. (1997a). Modality-specific processing streams in verbal working memory: evidence from spatio-temporal patterns of brain activity. *Cogn. Brain Res.* 6 95–113. 10.1016/s0926-6410(97)00021-9 9450603

[B64] Ruchkin D. S. Johnson R. Grafman J. Canoune H. Ritter W. (1997b). Multiple visuospatial working memory buffers: evidence from spatiotemporal patterns of brain activity. *Neuropsychologia* 35 195–209. 9025123 10.1016/s0028-3932(96)00068-1

[B65] Ruchkin D. S. Canoune H. L. Johnson R. Ritter W. (1995). Working memory and preparation elicit different patterns of slow wave event-related brain potentials. *Psychophysiology* 32 399–410. 10.1111/j.1469-8986.1995.tb01223.x 7652117

[B66] Ruchkin D. S. Johnson R. Canoune H. Ritter W. (1990). Short-term memory storage and retention: an event-related brain potential study. *Electroen. Clin. Neuro.* 76 419–439. 10.1016/0013-4694(90)90096-3 1699736

[B67] Ruchkin D. S. Johnson R. Grafman J. Canoune H. Ritter W. (1992). Distinction and similarities among working memory processes: an event related potential study. *Cogn. Brain Res.* 1 53–66. 10.1016/0926-6410(92)90005-C 15497435

[B68] Saling L. L. Phillips J. G. (2007). Automatic behaviour: efficient not mindless. *Brain Res. Bull.* 73 1–20. 10.1016/j.brainresbull.2007.02.009 17499631

[B69] Singhal A. Culham J. C. Chinellato E. Goodale M. A. (2007). Dual-task interference is greater in delayed grasping than in visually guided grasping. *J. Vis.* 7 1–12. 10.1167/7.5.5 18217845

[B70] Smyth M. M. Pearson N. A. Pendleton L. R. (1988). Movement and working memory: patterns and positions in space. *Q. J. Exp. Psychol.* 40 497–514. 10.1080/02724988843000041 3175032

[B71] Smyth M. M. Pendleton L. R. (1989). Working memory for movements. *Q. J. Exp. Psychol.* 41 235–250. 10.1080/14640748908402363 2748930

[B72] Spiegel M. A. Koester D. Schack T. (2013). The functional role of working memory in the (re-) planning and execution of grasping movements. *J. Exp. Psychol.-Hum. Percept. Perform.* 39 1326–1339. 10.1037/a0031398 23339349

[B73] Spiegel M. A. Koester D. Schack T. (2014). Movement planning and attentional control of visuospatial working memory: evidence from a grasp-to-place task. *Psychol. Res.* 78 494–505. 10.1007/s00426-013-0499-3 23832553

[B74] Spiegel M. A. Koester D. Weigelt M. Schack T. (2012). The costs of changing an intended action: movement planning, but not execution, interferes with verbal working memory. *Neurosci. Lett.* 509 82–86. 10.1016/j.neulet.2011.12.033 22230898

[B75] Studer P. Wangler S. Diruf M. S. Kratz O. Moll G. H. Heinrich H. (2010). ERP effects of methylphenidate and working memory load in healthy adults during a serial visual working memory task. *Neurosci. Lett.* 482 172–176. 10.1016/j.neulet.2010.07.030 20643186

[B76] Tucker M. Ellis R. (2001). The potentiation of grasp types during visual object categorization. *Vis. Cogn.* 8 769–800. 10.1080/13506280042000144

[B77] Tucker M. Ellis R. (2004). Action priming by briefly presented objects. *Acta Psychol.* 116 185–203. 10.1016/j.actpsy.2004.01.004 15158182

[B78] van Schie H. T. Bekkering H. (2007). Neural mechanisms underlying immediate and final action goals in object use reflected by slow wave brain potentials. *Brain Res.* 1148 183–197. 10.1016/j.brainres.2007.02.085 17412310

[B79] Voelcker-Rehage C. Alberts J. L. (2007). Effect of motor practice on dual-task performance in older adults. *J. Gerontol. B Psychol. Sci. Soc. Sci.* 62 141–148. 10.1093/geronb/62.3.P141 17507581

[B80] Weigelt M. Rosenbaum D. A. Huelshorst S. Schack T. (2009). Moving and memorizing: motor planning modulates the recency effect in serial and free recall. *Acta Psychol.* 132 68–79. 10.1016/j.actpsy.2009.06.005 19591968

[B81] Westerholz J. Schack T. Koester D. (2013). Event-related brain potentials for goal-related power grips. *PLoS ONE* 8:e68501 10.1371/journal.pone.0068501 PMC3699524 23844211

[B82] Westerholz J. Schack T. Schütz C. Koester D. (2014). Habitual vs. non-habitual manual actions: an ERP study on overt movement execution. *PLoS ONE* 9:e93116 10.1371/journal.pone.0093116 PMC3972190 24691654

[B83] Wickens C. D. (2008). Multiple resources and mental workload. *Hum. Factor* 50 449–455. 10.1518/001872008X288394 18689052

[B84] Woodman G. F. (2010). A brief introduction to the use of event-related potentials in studies of perception and attention. *Atten. Percept. Psychophys.* 72 2031–2046. 10.3758/APP.72.8.2031 21097848 PMC3816929

