# Supplementary figures and images for: Movement Interferes with Visuospatial Working Memory during the Encoding: An ERP Study
Source: Front Psychol. 2017 May 29;8:871. doi: 10.3389/fpsyg.2017.00871 (PMC5447076; doi:10.3389/fpsyg.2017.00871)

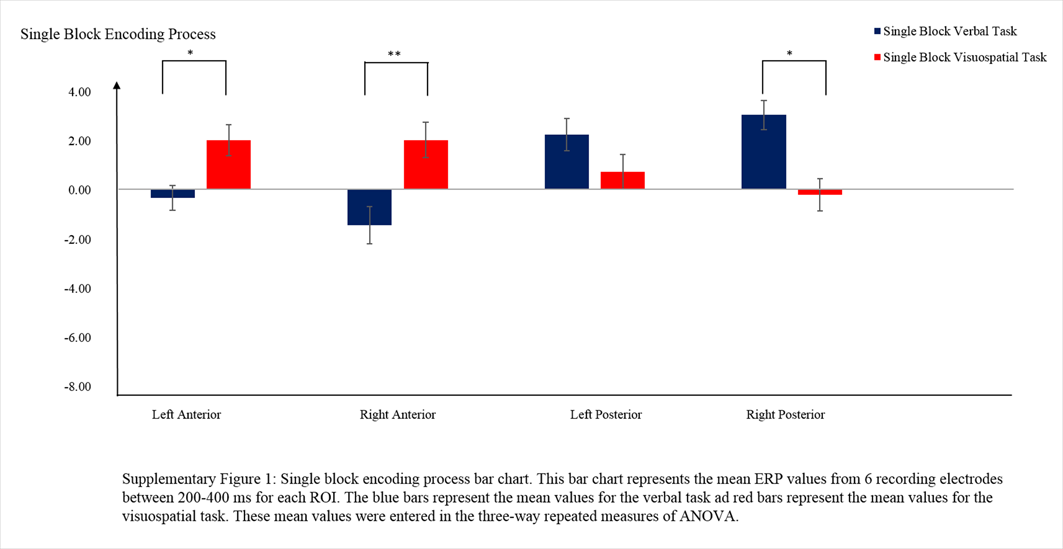

Supplement: Supplementary file 1 [file Image_1.TIF]

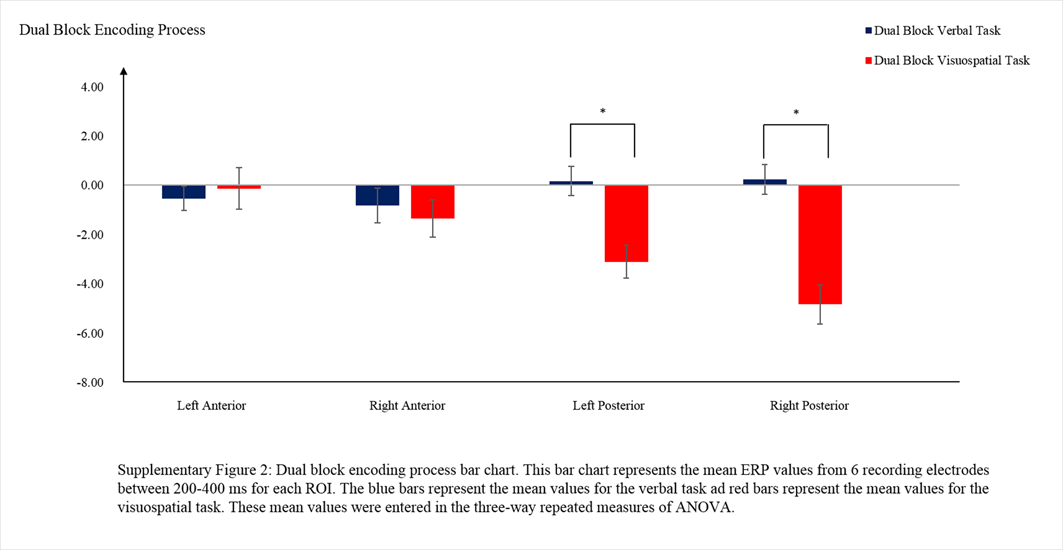

Supplement: Supplementary file 2 [file Image_2.TIF]

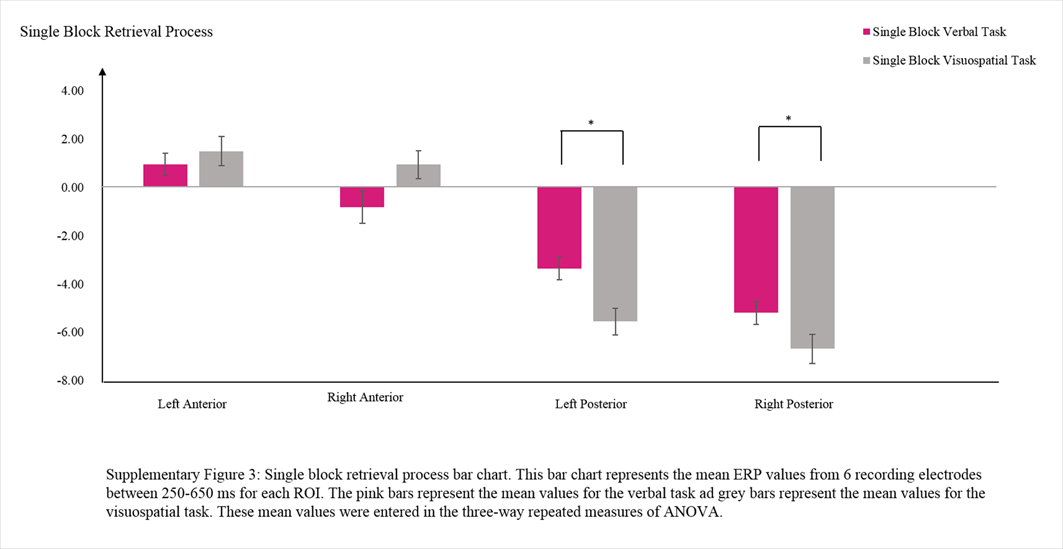

Supplement: Supplementary file 3 [file Image_3.TIF]

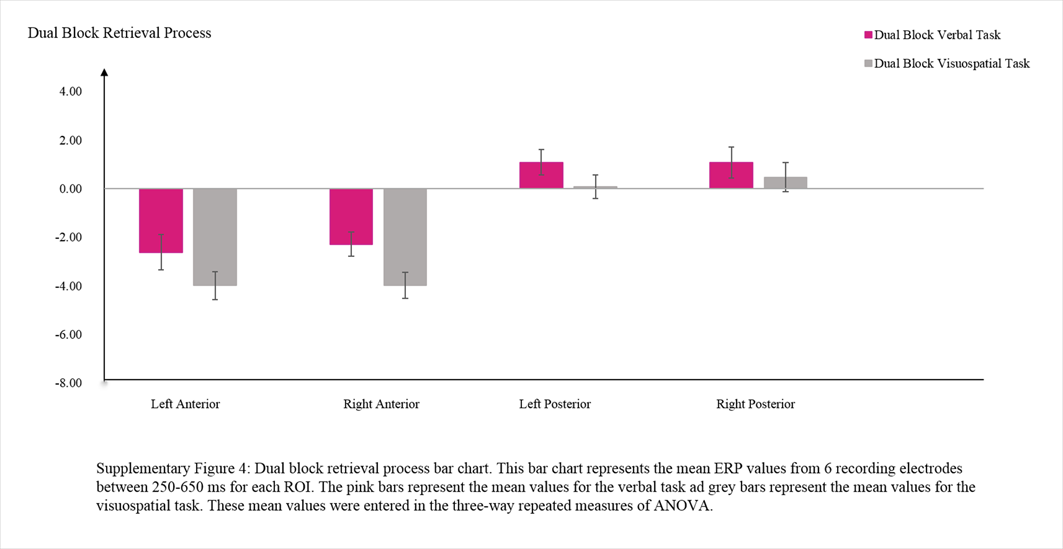

Supplement: Supplementary file 4 [file Image_4.TIF]

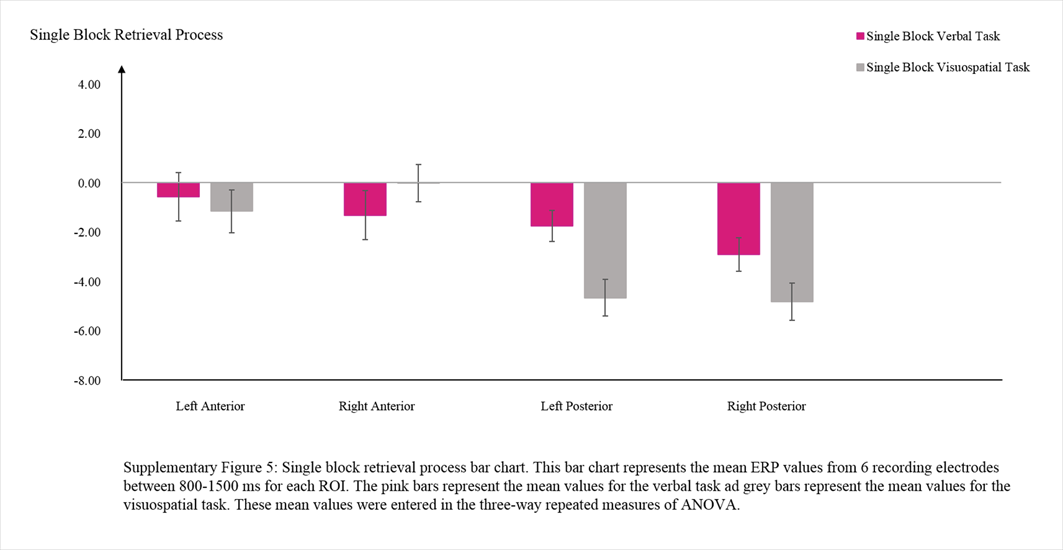

Supplement: Supplementary file 5 [file Image_5.TIF]

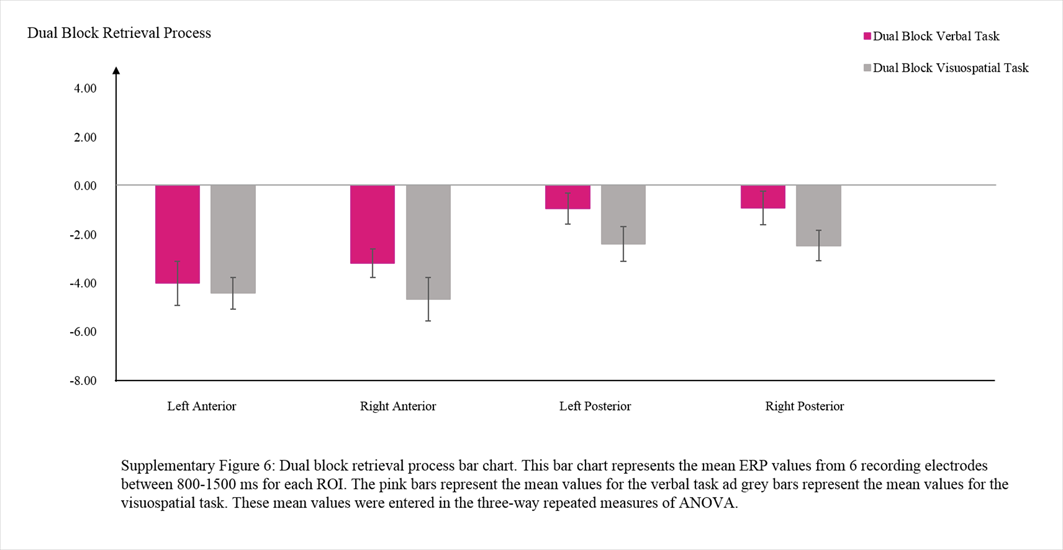

Supplement: Supplementary file 6 [file Image_6.TIF]
